# Innovative delivery systems for epicutaneous immunotherapy

**DOI:** 10.3389/fimmu.2023.1238022

**Published:** 2023-08-22

**Authors:** Zhen Wang, Lingzhi Wu, Wei Wang

**Affiliations:** ^1^Department of Pharmacy, The First Hospital of Jiaxing, First Affiliated Hospital of Jiaxing University, Jiaxing, China; ^2^College of Materials and Textile Engineering, Jiaxing University, Jiaxing, China

**Keywords:** epicutaneous immunotherapy, delivery systems, stratum corneum barrier, Viaskin, precise laser epidermal system, microneedles, nanocarriers

## Abstract

Allergen-specific immunotherapy (AIT) describes the establishment of peripheral tolerance through repeated allergen exposure, which qualifies as the only curative treatment for allergic diseases. Although conventional subcutaneous immunotherapy (SCIT) and sublingual immunotherapy (SLIT) have been approved to treat respiratory allergies clinically, the progress made is far from satisfactory. Epicutaneous immunotherapy (EPIT) exploits the skin’s immune properties to modulate immunological response, which is emerging as a promising alternative and has shown effectiveness in many preclinical and clinical studies for both respiratory and food allergies. It is worth noting that the stratum corneum (SC) barrier impedes the effective delivery of allergens, while disrupting the SC layer excessively often triggers unexpected Th2 immune responses. This work aims to comprehend the immunological mechanisms of EPIT, and summarize the innovative system for sufficient delivery of allergens as well as tolerogenic adjuvants. Finally, the safety, acceptability, and cost-effectiveness of these innovative delivery systems are discussed, which directs the development of future immunotherapies with all desirable characteristics.

## Introduction

Immunoglobulin E (IgE)-mediated allergies affect more than 30% of the population worldwide that brings a considerable medical and socioeconomic burden (1). Current allergen-specific immunotherapy (AIT) describes the establishment of peripheral tolerance through repeated allergen exposure, which qualifies as the only curative treatment for allergic diseases (2, 3). Conventional AITs, such as subcutaneous immunotherapy (SCIT) and sublingual immunotherapy (SLIT) have been approved to treat allergic patients, but they require frequent dosing over long-term, and often provoke undesirable systemic or local reactions (4, 5). Therefore, only <5% of allergy sufferers choice AIT as second-line treatment. A critical need remains safety, efficacy and acceptability of AIT, which is largely dependent on how the allergen is presented to immune system, emphasizing the innovative route and form of allergen administration (6, 7).

Epicutaneous immunotherapy (EPIT) exerts tolerogenic effects by applying allergens topically on intact or pre-treated skin. Within the epidermis and dermis, allergens are taken up by skin antigen presenting cells (APCs), such as Langerhans cells (LCs) and dermal dendritic cells (DCs) (8, 9), subsequently migrate through the dermis to local draining lymph nodes (LNs), where they can elicit T cell polarization and tolerance (10). The first study of successful EPIT dates back to 1921, and it was observed that applying allergens onto scarified skin reduced allergic symptoms in patients with a horse allergy (11). The development of tolerogenic effects is mediated by CD4^+^ CD25^+^ Foxp3^+^ Tregs and secreting TGF-β. Mouse experiments showed that Tregs required CTLA-4 surface marker expression but not IL-10 (12). Both humoral and T-cellular effects have been noticed after EPIT, including the generation of Tregs, the induction of specific IgG2a and a diminished Th2 immune response (13).

EPIT has shown varying degrees of success in phase II/III clinical trials (13, 14), and simultaneously satisfies the urgent need of needle-free and self-administration. Since allergens are introduced into epidermis, the non-vascularized skin layer can lower the antigen leakage into systemic circulation and minimize the risk of adverse events, promoting the clinical medication safety (14–16). Moreover, EPIT fully exploits the skin’s innate immune properties, and the high density of APCs in epidermis and dermis implies smaller amounts of antigen to activate immune response, compared to subcutaneous injections (17, 18). However, the exist of stratum corneum (SC) impedes epidermal delivery of allergen *via* intact skin, whereas damaging this barrier often results in unwanted Th2 responses (19, 20). Thus, the desirable EPIT must keep a balance between skin disruption and allergen delivery. In this work, we review the immunological mechanisms, and summarize the innovative delivery system for allergens as well as tolerogenic adjuvants in preclinical and clinical EPIT (**Tables 1**, **2**). The safety, acceptability, and cost-effectiveness of these novel systems are appraised (**Table 3**), which directs the development of future immunotherapies with all desirable characteristics.

**Table 1:** List of EPIT clinical trials.

Years	Areas	Phase	Sample sizes	Ages	Diagnosis	Intervention	Duration	Outcomes	Reference
2006-2007	Switzerland	1, 2	37	18-65	Grass pollen-induced rhinoconjunctivitis	Tape-stripped + Patch Phl p 5 (21 μg)	4-5 months	SS, RM, NPT, SPT, L-TEAE, S-TEAE, SAE	(21)
2008-2009	Switzerland	1, 2	132	18-65	Grass pollen-induced rhinoconjunctivitis	Tape-stripped + Patch Phl p 5 (3, 15, 30 μg)	4-5 months	SS, RM, CPT, SPT, HEP, L-TEAE, S-TEAE, SAE	(22) NCT00719511
2008-2010	Switzerland	1, 2	97	18-65	Grass pollen-induced rhinoconjunctivitis	Tape-stripped + Patch Phl p 5 (21 μg)	4-5 months	SS, RM, CPT, SPT, HEP, L-TEAE, S-TEAE, SAE	(23) NCT00777374
2010-2012	USA	1b	100	6-50	Peanut allergy	Viaskin peanut (20, 100, 250, 500 μg)	2 weeks	sIgE, RM, TEAE, L-TEAE, SAE, SCORAD	(24) NCT01170286
2010-2015	France	2	54	5-17	Peanut allergy	Viaskin peanut (20, 100, 250, 500 μg)	12 months	OFC, SPT, sIgE, sIgG4, TEAE, L-TEAE, SAE	NCT01197053
2012-2014	Europe and North America	2b	221	6-55	Peanut allergy	Viaskin peanut (50, 100, 250 μg)	24 months	CRD, OFC, SPT, sIgE, sIgG4, TEAE, L-TEAE, SAE	(25) NCT01675882
2013-2018	USA	2	74	4-25	Peanut allergy	Viaskin peanut (100, 250 μg)	52 weeks	OFC, SPT, sIgE, sIgG4, TEAE, L-TEAE, SAE, SCORAD	(26) NCT01904604
2013-2016	Europe and North America	2b	171	7-56	Peanut allergy	Viaskin peanut (250 μg)	36 months	CRD, OFC, SPT, sIgE, sIgG4, TEAE, L-TEAE, SAE	NCT01955109
2014-2017	North America	2	198	2-17	Milk allergy	Viaskin milk (150, 300, 500 μg)	12 months	OFC, SS, SPT, sIgE, sIgG4, TEAE, L-TEAE, S-TRAE, SAE	NCT02223182
2015-2018	USA	2a	20	4-11	Milk-induced Eosinophilic Esophagitis	Viaskin milk (500 μg)	11 months	OFC, SS, L-TEAE, S-TEAE, SAE	(27) NCT02579876
2015-2017	Europe and North America	3	356	4-11	Peanut allergy	Viaskin peanut (250 μg)	12 months	CRD, OFC, sIgE, sIgG4, TEAE, L-TEAE, S-TRAE, SAE	(28, 29) NCT02636699
2015-2019	Europe and North America	3	198	4-11	Peanut allergy	Viaskin peanut (250 μg)	36 months	CRD, OFC, sIgE, sIgG4, TEAE, L-TEAE, S-TEAE, SAE	(30) NCT03013517
2017-2023	USA	3	362	1-3	Peanut allergy	Viaskin peanut (250 μg)	12 months	CRD, OFC, sIgE, sIgG4, TEAE, L-TEAE, S-TEAE, SAE	(31) NCT03211247
2023- (recruiting)	North America	3		4-7	Peanut allergy	Viaskin peanut (250 μg)	12 months	OFC, SPT, sIgE, TEAE, L-TEAE, SAE, SCORAD	NCT05741476

CPT, Conjunctival provocation test; CRD, Cumulative reactive dose; HEP, Histamine equivalent prick; NPT, nasal provocation test; OFC, oral food challenge; RM, rescue medication; SS, symptoms scores; SCORAD, Scoring Atopic Dermatitis; SPT, Skin prick test; sIgE, specific immunoglobulin E; sIgG, specific immunoglobulin G; TEAE, Treatment-emergent adverse events; L-TEAE, local treatment-emergent adverse event; S-TEAE, systemic treatment-emergent adverse event; SAE, serious adverse events.

**Table 2:** Recent advances in EPIT studies.

Study model	Delivery system	Allergen	Adjuvant	Duration	Outcomes	Reference
Balb/c	P.L.E.A.S.E.	HDM (100 μg)		Once weekly for 8 weeks	HDM-specific IgG ↑, with comparable IgG levels to SCIT HDM-specific IgE ↓, with lower IgE levels than SCIT Th2 cytokines (IL-4, IL-5, IL-13) in splenocytes↓ FoxP3^+^ T cells in the BALF ↑ Penh values ↓	(32)
Balb/c	P.L.E.A.S.E.	Phl p 5 (50 μg)	CpG (100 μg)	Twice weekly for 3 weeks	Th2 immune response with Phl p 5 only Th1 immune response with CpG addition Phl p 5-specific IgG2a ↑, Phl p 5-specific IgE ↓ IL-4, IL-5, IL-13 in splenocyte culture supernatants ↓	(33)
Balb/c	P.L.E.A.S.E.	OVA (50 μg)	CpG (5 μg), VD_3_ (10 ng)	Once weekly for 3 weeks	OVA-specific serum IgG2a ↑, IgE ↓ Infiltration of eosinophils and neutrophils into the lung and BALF ↓ Average airway wall thickness ↓ OVA-specific Treg cells in spleen ↑	(34)
Balb/c	P.L.E.A.S.E.	LamOVA (86 μg)		Once weekly for 8 weeks	OVA-specific serum IgG ↑, equally effective as SCIT OVA-specific serum IgE ↓, lower than SCIT Th2 cytokines in splenocyte culture supernatants ↓, lower than SCIT Lung inflammation ↓ No local side effects	(18)
Balb/c	Coated Microneedles	Peanut (5 μg)		Single patch applied for 3 min, once weekly for 3 weeks	Peanut-specific serum IgG, IgG1 and IgG2a↑, peanut-specific serum IgE↓ IL-2 and IFN-γ↑, IL-4 and IL-5↓ in splenocyte culture supernatants Clinical anaphylaxis symptom score and temperature drop upon oral challenge ↓ Mast cell protease 1 (MCP-1) and histamine upon oral challenge↓ Eosinophil infiltration in small intestine↓	(35)
C3H*/*HeJ	Coated Microneedles	Peanut (11 μg)		Single patch applied for 5 min, once weekly for 5 weeks	Peanut-specific serum IgG2a and IgG2b↑, IgE↓ IL-10 and IFN-γ↑, IL-4, IL-5, IL-21 ↓ in spleen and mesenteric lymph node Clinical anaphylaxis symptom score and temperature drop upon oral challenge ↓ MCP-1 upon oral challenge↓ Ear swelling and SPT wheal diameter upon intradermal challenge ↓ Eosinophil and mast cell infiltration in the intestine ↓	(36)
Balb/c and C57BL/6J	Dissolving Microneedles	Peanut (12 μg)	CpG (0.12 μg), VD_3_ (1.2 ng)	Two patches applied for 1 h, once weekly for 6 weeks	Peanut-specific serum IgG2a↑, peanut-specific serum IgE ↓ FoxP3^+^ Tregs in lymph nodes and spleens↑ Clinical symptom score ↓ MCP-1, mast cells, and eosinophils in intestinal jejunum ↓	(37)
Balb/c	Biodegradable Microneedles	Der f 1 (10 μg)		Single patch applied for 2 h, twice weekly for 4 weeks	HDM-specific IgG2a ↑, IgE ↓ Eosinophils, macrophages and neutrophils in BALF↓ IL-10, TGF-β and IFN-γ in lungs ↑ IL-4, IL-5, IL-13, IL-33 and TSLP in lungs ↓ Mucus hyperplasia and subepithelial fibrosis in lungs ↓	(38)
Balb/c	Coated Microneedles	Der p 1 (25 μg)	CpG (25 μg)	Single patch applied for 3 min, once weekly for 3 weeks	Der p 1-specific serum IgG, IgG1 and IgG2a ↑ IL-10, TGF-β and IFN-γ in BALF ↑, IL-5 and IL-13 in BALF ↓ IL-2 and IFN-γ ↑, IL-4, IL-5 and IL-13 ↓ in splenocyte culture supernatants Hydroxyproline in BALF ↓ Infiltration of macrophages and neutrophils in BALF ↓ Infiltration of eosinophils and mast cells in lungs ↓ Mucus deposition in bronchioles ↓	(39)
Balb/c	Coated Microneedles	OVA (25 μg)	CpG ODN 1826 (25 μg)	Single patch applied for 3 min, once weekly for 3 weeks	Serum and BALF levels of OVA-specific IgG, IgG1, IgG2a↑ IL-10 in BALF ↑, IL-4, IL-5 and IL-13 in BALF↓ IFN-γ and IL-2 ↑, IL-4 and IL-13↓ in splenocyte culture supernatant Infiltration of neutrophils, macrophages and mast cells ↓ Mucus hyperplasia, collagen deposition around lung bronchioles ↓	(40)
Balb/c	Coated Microneedles	OVA (25 μg)	CpG ODN 1826 (25 μg)	Single patch applied for 3 min, once weekly for 3 weeks	OVA-specific serum IgG1 and IgG2a ↑ IL-10 in BALF ↑, IL-5 and IL-13 in BALF↓ Infiltration of mast cells and eosinophils in the lung↓ Mucus hyperplasia inside the lung bronchioles ↓	(41)
C3H*/*HeJ	S/O nanodispersion	β-lactoglobulin (25 μg)	R848 (2.5 μg)	Single patch applied for 24 h, once weekly for 3 weeks	Specific serum IgG1 and IgG2a ↑, compared with SCIT IFN-γ, IL-12p40 and IL-10↑, IL-4 and IL-13↓ in splenocyte culture supernatant Ear thicknesses of after skin contact ↓	(42)
Balb/c	S/O nanodispersion	7CrpR (25 μg)	R848(2.5 μg)	Single patch applied for 48 h, once weekly for 3 weeks	Specific serum IgE and total IgE ↓ Addition of the R848 shifted the Th1/Th2-balance toward Th1-type immunity	(43)
Balb/c	TPP, aptamer, GNP	rChe a 2 (10, 5, 2.5 μg)		Single patch applied for 48 h, once weekly for 6 weeks	Serum IgE ↓ IFN-γ, TGF-β, and IL-10 ↑, IL-4, and IL-17a ↓ in splenocyte culture supernatants Eosinophil cell counts in NALF ↓ SPPs led to more significant improvement of IL-10	(44)

**Table 3:** Advantages and limitations of delivery systems for EPIT.

System	Allergen	Adjuvant	Advantages	Limitations	Duration
Viaskin	Peanut, milk, OVA, HDM, pollen		A high safety profile due to the allergen application onto non-vascularized epidermis More convenient and compliant for the patients by a non-invasive and self-administrable application method No serious adverse event or epinephrine use during treatment No visible, long-lasting damage to skin Free of additional irritant constituents (e.g. alum, or preservatives) Less cost intensive than conventional AIT	Low rates of allergen delivery into the epidermis Limited to large molecule drugs Prolonged patch application times Less consistent outcomes across different populations Moderate efficacy in clinical trials More data are needed regarding aeroallergens	Single patch applied for 20-24 h daily, 1-3 years in clinical trials Single patch applied for 48 h, once weekly for 8 weeks in mice models
P.L.E.A.S.E.	HDM, Phl p 5, OVA	CpG, VD_3_	The high diffusion rate of biomacromolecule *via* laser-generated micropores Needle-free and painless application Improved safety and optimal patient compliance Selective ablation of epidermal and dermal layers	Inconvenience and high cost Local side effects (itching, eczema) and temporary hyperpigmentation	3-8 times in mice models
Microneedles	Peanut, Der f 1, Der p 1, OVA	CpG	Large molecules can be administered Painless, needle-free and self-administrable application No visible, long-lasting damage to skin Room temperature storage	Limited drug loading Less immunogenicity with repeated dissolving and drying Local and systemic allergic reactions	Patch applied for 3-120 min, total 3-8 times in mice models
Nanosystems	β-lactoglobulin, 7CrpR, rChe a 2, Api m 1	R848	Loaded with both antigen and adjuvant Controlled release behaviors Similar in size to the pathogens The increased APC-particle interactions after modification Protecting the antigen until reaching the inside of the targeted cell	Limited to biomacromolecules Prolonged application times Less understanding on the physicochemical properties of nanosystems More data are needed between nanoparticles and immune system Difficult in large-scale synthesis	Single patch applied for 24-48 h, 3-6 weeks in mice models

## The Viaskin epicutaneous delivery system

**Figure 1:**
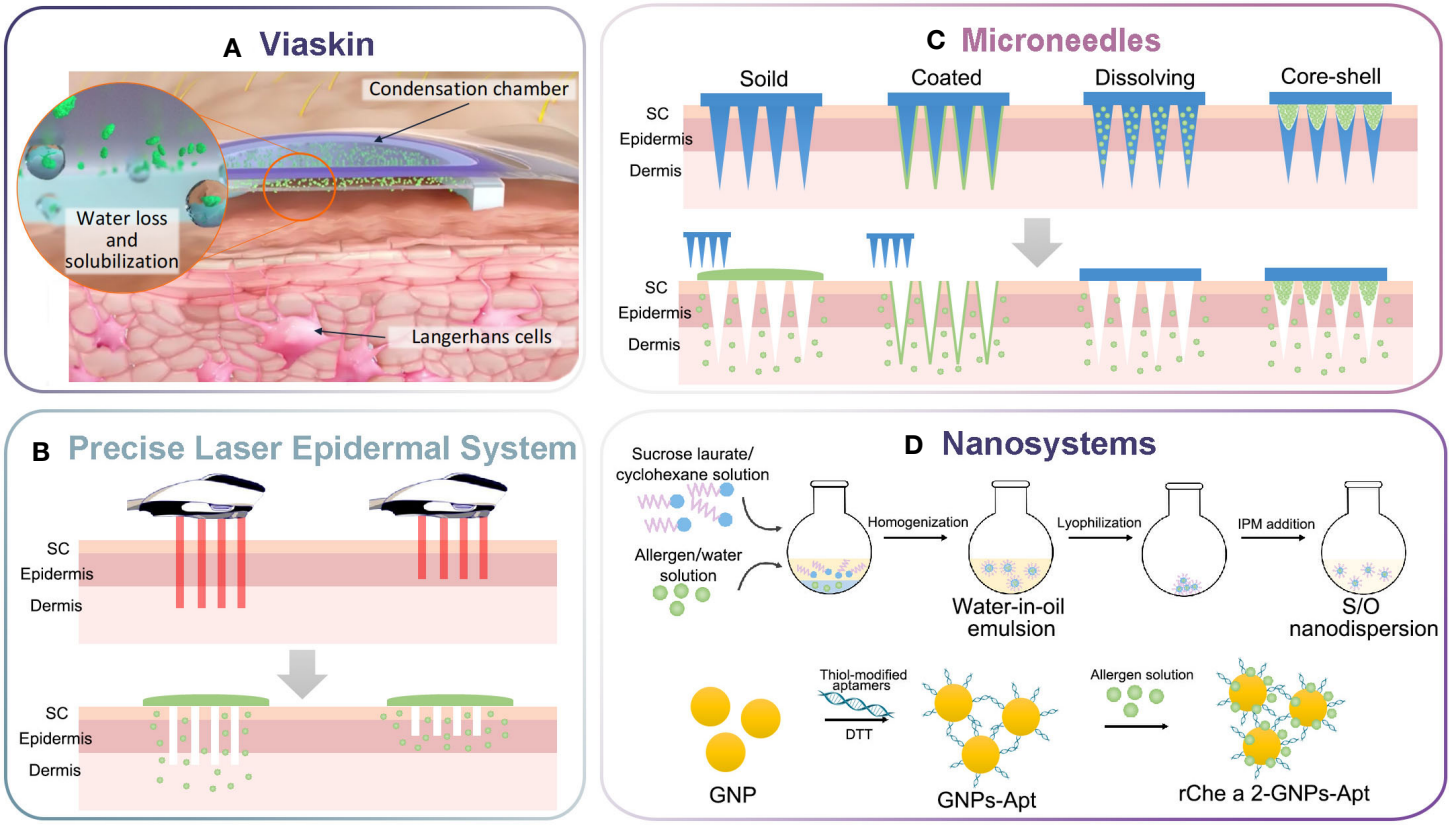
Schematic illustration of innovative delivery systems for EPIT. **(A)** Viaskin system (https://dbv-technologies.com). **(B)** Precise laser epidermal system. **(C)** Microneedle delivery system. **(D)** Nano-drug delivery system.

Initially, the skin was prepared by adhesive tape-stripping to remove SC and enhance solvent allergen delivery, whereas the majority of adults reported local reactions and systemic Th2 response, with more than 70% eczema at the treated area (21–23). To minimize Th2 immune responses, EPIT is performed onto intact skin through Viaskin epicutaneous delivery system (DBV Technologies, France) in most preclinical and clinical trials (14). It is engineered by electrostatic spraying powdered allergens onto a transparent plastic membrane. When applied on the skin, Viaskin creates an enclosed chamber and utilizes transepidermal water loss to increase permeability of SC (**Figure 1A**) (13, 17).

Mondoulet and Dioszeghy group evaluated Viaskin system in mouse models with respiratory and food allergies. In mice sensitized to house dust mite (HDM), pollen, ovalbumin (OVA) or peanut, EPIT had equivalent curative effect to SCIT, considered as the reference immunotherapy (45). The repeated applications of Viaskin patches should be performed on intact skin to insure the safety and efficacy, but not stripped skin (20). What was more, EPIT was at least as efficient as SLIT in mice sensitized to Phleum pratense pollen (46). The EPIT-induced Tregs were still effective eight weeks after the end of treatment, whereas that in SLIT lost their suppressive activities (12). In peanut-sensitized mice, the Tregs induction by EPIT also mediated long-term protection from eosinophilic disorders (47).

The characteristics of EPIT trials were summarized in **Table 1**. The Viaskin patches were preferentially developed for the treatment of food allergies. In phase I study (NCT01170286), the safety and tolerability of Viaskin peanut were evaluated at doses of 20, 50, 200 or 500 μg, as well as placebo. The localized treatment-emergent adverse events (L-TEAE) that emerged during the study were primarily mild to moderate, affecting 86.3% of peanut-treated patients compared to 60% of placebo-treated individuals. Peanut EPIT through Viaskin system on intact skin was safe and well tolerated, with high adherence by participants (24). Subsequent phase II studies were conducted to evaluate therapy efficacy and dose variation effect on adults or children. Both studies demonstrated significant absolute difference in response rates of 53.6% in children aged 6-11, and 38.9% in adolescents aged 12-17, with daily 24 h application (250 µg) for 12 months (25). The compliance rate was more than 97% across all cohorts. However, adults showed no significant response to treatment. To assess the efficacy and adverse events further, a phase 3, randomized, double-blind, placebo-controlled trial was conducted with children aged 4-11 years (NCT02636699). Under daily treatment with Viaskin peanut (250 µg) for 12 months, the responder rate was found to be 35.3% in the peanut group, compared to only 13.6% with placebo, but did not meet the prespecified lower bound of the confidence interval criterion for a positive trial result (28). The incidence rate of TEAE was 95.4% in peanut group vs 89% in placebo group, and all of them were mild or moderate severity. Then, subjects who successfully completed the 12-months study were enrolled in an additional 2 years with daily treatment (NCT03013517). At month 36, 75.9% of patients demonstrated increased eliciting dose compared with baseline, and 51.8% of subjects reached an eliciting dose of ≥1000 mg, compared with 40.4% at month 12. There was no treatment-related epinephrine use during treatment. The compliance for Viaskin was high (96.9%), and withdrawals were lower than 1% due to TEAE. For children younger than 4 years of age, a phase 3 trial was also carried out (NCT03211247). The primary efficacy end point result was observed in 67.0% of toddlers in the intervention group, compared to 33.5% with placebo (31). Additionally, Viaskin has been used on intact skin to treat children with cow’s milk allergy and milk-induced eosinophilic esophagitis (23, 27, 48).

Viaskin-mediated EPIT successfully increases tolerance for food allergy without any serious anaphylaxis incidents during study, suggesting the advantages of high safety and evident efficacy. The comparison of efficiency between EPIT and SCIT in clinical is not available, because SCIT for food allergy has been abandoned given the significant rate of severe, systemic reactions. Similarly, there is no head-to-head comparison of Viaskin-EPIT to other routes of administration, except in mouse models. What is more, Viaskin-EPIT is easy to use with minimal restrictions on daily activities, resulting in high compliance rates. However, the SC poses a major barrier, and passive diffusion of allergens through intact skin was less than 10% after 24 h application (49). It can explain the moderate efficacy in clinical trials, whereas the prolonged application leads to significant skin irritation. On the other hand, the morphological differences of skins might cause less consistent outcomes across different populations (25). This suggests that responses to Viaskin-mediated EPIT might be more robust in younger patients, so the phase 3 trial only limits to children (26). Moreover, Viaskin patch is restricted to deliver allergen powders only, so it is challenging to introduce tolerogenic adjuvants in the system.

## Precise laser epidermal system

Ablative fractional laser (AFL) creates microchannels in epidermal or dermal layers to enhance transcutaneous drug delivery, particularly for biomacromolecules (**Figure 1B**). It targets hydrophilic cutaneous tissue and emits energy to explosive evaporation of water, which creates aqueous micropores (about 50-150 µm in diameter) in the skin with minimized thermal tissue damage (50). Subsequently, the aqueous solution or powder of allergens are applied onto the created micropores. The commercially available precise laser epidermal system (P.L.E.A.S.E.) allow to vary the density and depth of micropores, resulting from the adjustable parameters such as the number of microchannels per surface area, number of pulses per microchannel (51). Unlike tape-stripping, the P.L.E.A.S.E. device achieve selective ablation of epidermal and dermal layers, enabling more individualized skin targeting (52).

In early studies, the aqueous solution of OVA and Phl p 5 were applied onto the AFL-treated skin. Immunizations with OVA or Phl p 5 led to a distinct Th2 immune response, while the CpG addition manipulated it towards Th1 milieu, with increased IgG2a secretion and decreased IgE, IL-4, IL-5 and IL-13 (33, 53). We inferred that micropores with depths of 30-40 µm went through the epidermis to basement membrane, and the allergen exposure to dermis might induce sensitization (17). Alternatively, Korotchenko et al. created micropores with well-defined depth and density using P.L.E.A.S.E. device to facilitate the delivery and uptake of topically applied allergens, which was emerging as an alternative to classical SCIT or SLIT for HDM-induced lung inflammation (32).

The powder delivered within the microchannels could be hydrated by interstitial fluid, then dissolved and spread over the epidermis with minimal leakage to circulating system, whereas aqueous allergens diffused quickly (34). As captured by confocal microscopy, the GFP^+^ APCs were attracted and accumulated around individual microchannel. It increased rapidly and reached the peak on 3 days, and declined over 6 to 10 days (54, 55). Motivated by these hypotheses, Kumar et al. used P.L.E.A.S.E. to generate micropores with a size of 50-75 μm in base diameter and 20-30 μm in depth, then a mixture powder of OVA, 1,25-Dixydroxyvitamin D_3_ (VD_3_), and CpG was applied onto the microchannels for 2 h. The topical application promoted high levels of epidermal delivery, and the retained powder was beneficial to create an “antigen-depot” effect and stimulated the immune system continuously for a long time. Only three times of EPIT significantly suppressed airway hyperresponsiveness and lung inflammation of OVA-sensitized mice, which was unattainable by SCIT. The EPIT with OVA-CpG-VD_3_ induced higher Tregs, and favored IgG2a expression from Th1-biased immune response (34).

Enhanced and controlled activation of DC may be achieved through the specific targeting of surface receptors such as C-type lectin receptors. The P.L.E.A.S.E. device allows for effective delivery of DC-targeted allergens into the epidermis, thereby increasing the immune protection and reducing side effects. In a study by Korotchenko et al., laminarin-ovalbumin neoglycoconjugates (LamOVA) were synthesized using a 2-step reductive amination method, followed by analysis of their immunogenicity, allergenicity, and therapeutic efficacy. Laminarin conjugation to OVA significantly facilitated uptake by bone marrow-derived dendritic cells (BMDCs), leading to their activation. The LamOVA conjugates showed a five-fold reduction in IgE binding capacity, while their immunogenicity increased to three-fold. What was more, EPIT with LamOVA induced higher IgG and suppressed lung inflammation, which was equally effective as SCIT, although the latter was associated with elevated IgE and Th2 cytokines (18). Similarly, mannan conjugates are known to enhance allergen uptake by skin DCs, particularly LCs and CD14^+^ dermal DCs (56). For instance, Mannan-Bet v 1 was synthesized using mild oxidation and reductive amination, then applied to P.L.E.A.S.E.-treated mouse skin (57). The allergenicity of Mannan-Bet v 1 was about six orders of magnitude lower than that of soluble Bet v 1, making it less likely to trigger IgE-dependent responses. Furthermore, the same conjugation approach could be applied to hypoallergenic Phl p 5 (33).

Briefly, the precise laser epidermal system increased the quantity of antigen delivered into the epidermis without systemic passage. As a needle-free and painless approach, it has potential to replace standard methods due to the improved safety and optimal compliance. From a research point of view, the adaptable parameters will allow us to investigate the underlying immunological mechanisms and design the vaccination strategies rationally. Moreover, tailored DC targeting with polysaccharide conjugation improved uptake and increased the level of DC activation specifically (52). Despite the potential benefits of P.L.E.A.S.E. device in therapy, challenges such as inconvenience and high cost still need to be addressed before it can be widely practiced in self-administration.

## Microneedle delivery system

Microneedles have emerged as an attractive platform for transdermal or topical drug delivery, comprising solid, coated, dissolving, and hollow microneedles. These systems can pierce through SC layer perpendicularly, and selectively deliver the allergens and adjuvants into epidermis or dermis for uptake by skin APCs (58). Since the performance is not affected by skin permeabilities between children and adults, microneedles allow strict dose control, and reduce application time from 24 h to 3-5 min (59). This not only improves the reliability and compliance of immunotherapy but also renders them painless, non-invasive, safe, and tolerable across all age groups (60). The following sections highlights the microneedles as an innovative delivery system in EPIT (**Figure 1C**).

In earlier studies, solid microneedles were employed to penetrate the skin resembling AFL, followed by topically allergens application. However, these studies were not involved in sensitized animal models. Coated microneedles were introduced next, in which the allergen was coated on the surface and rapidly dissolved in the aqueous environment of skin tissue, allowing for freely delivery of allergens into epidermis/dermis. Studies by Gill group utilized stainless-steel, dip-coated microneedles for the treatment of respiratory and food allergies in mouse models (35, 36, 39–41). In a pioneering study, the peanut protein-coated microneedles (5 μg) were performed once weekly for 3 weeks in peanut-sensitized mice (35). After oral challenge, clinical symptoms of peanut-induced anaphylaxis were significantly reduced following treatment, accompanied with the higher IgG1 and IgG2a. The analysis of splenocyte culture supernatants showed elevated levels of IL-2 and IFN-γ, but decreased levels of IL-4 and IL-5 compared to untreated group, indicating a Th1 dominant response. Landers et al. directly compared the therapeutic benefit of coated microneedles (11 μg) to EPIT (100 μg) in peanut sensitized mice and found that treatment with microneedles was safe and resulted in enhanced desensitization compared to EPIT (36). The mice with coated microneedles were better protected from body temperature drop and anaphylaxis symptom scores upon oral challenge, while those with EPIT exhibited allergic symptoms. These findings suggested that coated microneedles had the potential to deliver allergens into the skin, thereby improving immune modulation and efficacy.

The Gill group also evaluated both the therapeutic and prophylactic capabilities of coated microneedles in mouse models of airway allergy (39–41). In a comparison between microneedles (OVA + CpG) and SCIT (OVA + Alum), mice receiving OVA + CpG were found to induce higher levels of OVA-specific IgG1 compared to those treated with SCIT (41). The therapeutic capacity was evidenced by the suppression of airway inflammation upon intranasal OVA challenge, with reduction of eosinophils in the lung tissues, and low deposition of mucus inside lung bronchioles. Coated microneedles also demonstrated prophylactic efficacy to prevent the allergy progression of mice that were vaccinated with OVA + CpG, as confirmed by the regulated Th2 cytokines and anti-inflammatory cytokines in BALF (40). In another study, Gill et al. examined the effectiveness of microneedles in delivering Der p1 into mouse skin to prevent the development of Der p1-induced airway allergy (39). Post challenge, mice vaccinated with Der p 1 + CpG-coated microneedles showed a greater IgG2a response than the Der p 1-coated microneedles and SCIT groups, suggesting the beneficial role of CpG for allergy vaccination. Microneedles were minimally invasive, painless, and self-administration, making them a user-friendly alternative to SCIT. However, the system used in these studies were about 700 μm in length, which increased the possibility that allergen spreading in the dermis could reach the bloodstream and cause anaphylaxis.

The dissolving microneedles were demonstrated to avoid biohazardous wastes and promote drug loading (59). Park et al. designed Der f 1-loaded sodium hyaluronate microneedles using the droplet-born air blowing method, and compared their efficacy to conventional SCIT in murine asthma model (38). The Der f 1-loaded biodegradable microneedles alleviated airway hyperresponsiveness, eosinophilic infiltration, goblet cell hyperplasia and subepithelial fibrosis. These changes were more significant in the low-dose (10 μg) microneedle group than in high-dose (100 μg) SCIT group. In another study, a powder-laden, dissolving microneedle arrays (PLD-MNA) were engineered to deliver the aforementioned powder allergens into epidermis. The outer shell of PLD-MNA was made of highly biocompatible carboxymethyl cellulose, and the concave was filled directly with powder of peanut protein, VD_3_ and CpG. Notably, the immunogenicity of lyophilized allergens was fully preserved, while more than 50% immunogenicity could be lost with repeated dissolving and drying during the fabrication of coated and dissolving microneedles. After skin insertion, PLD-MNA deposited the powder in to epidermis with minimal leakage to the circulation system and attracted a large number of APCs (54). In the preclinical study, microneedle-mediated immunotherapy produced enhanced outcomes when compared to sham treatments, with lower peanut-specific serum IgE, and decreased infiltration of intestinal mucosal mast cells and eosinophils. PLD-MNA required only 6 treatments and one-fifth of therapeutic dose, with improved outcomes compared to 12 intradermal immunotherapies.

## Nano-drug delivery system

Nano-based drug delivery systems can interact with skin components in a way, offering an attractive alternative for overcoming the limited skin penetration of molecules (61). A variety of drug nanocarriers, such as lipid nanoparticles, organic-inorganic nanoparticles, dendrimers and micelles, have been developed for both topical and transdermal delivery of therapeutics. In this review, we focus specifically on the performance of nanosystems in EPIT.

The solid-in-oil (S/O) and gel-in-oil (G/O) nanodispersion was proposed as a means of improving the dispersibility of hydrophilic allergens into an oil phase, thereby breaking down the skin barrier and enhancing drug permeability (62). Initially, Kitaoka et al. incorporated CpG with OVA into S/O nanodispersion for transcutaneous immunization, and investigated the effects on Th1/Th2 immune balance (63). It was noting that the permeability of ~40 kDa proteins remained lower than that of small size after application (62, 64). Subsequently, β-lactoglobulin (BLG) nanoparticles were prepared and dispersed into oil isopropyl myristate (IPM) with the aid of Sucrose laurate L-195 (**Figure 1D**). The S/O nanodispersion enhanced the skin permeation of BLG into the dermis, and skewed the immune response toward Th1 immunity, indicating the amelioration of allergic symptoms. This effect was reinforced when resiquimod (R848) was included in IPM (42). Likewise, the T cell epitope peptide (7CrpR) was efficiently delivered *via* the S/O nanodispersion and decreased IgE levels in pollinosis model mice. The addition of immunomodulator R848 shifted the Th1/Th2-balance toward Th1-type immunity significantly, demonstrating potential for alleviating Japanese cedar pollinosis (43, 65).

Koushki et al. developed a targeted delivery system comprised of functionalized gold nanoparticles along with the DC-specific aptamers and rChe a 2 allergen (rChe a 2-GNPs-Apt) (**Figure 1D**) (44). Additionally, skin-penetrating peptides (SPP) were topically applied to enhance skin permeability and therapeutic efficacy. Results demonstrated that the rChe a 2-GNPs-Apt stimulated the secretion of IFN-γ, TGF-β, and IL-10 in splenocyte culture supernatants, reduced IgE, IL-4 and IL-17a levels, as well as eosinophil cell counts in nasal lavage fluid (NALF), compared to non-targeted groups. Besides, microemulsions have great potential as a protein-containing drug delivery system due to their natural penetration enhancement, thermodynamic stability, and solubilization capacity. Kiselmann et al. focused on the bee-venom phospholipase A2 (Api m 1) and created a skin-friendly microemulsion *via* Phase Diagram *via* Micro Plate Dilution (PDMPD)-method, allowing the model allergen to penetrate epidermal layers and reach LC and dDC. However, subsequent *in vivo* studies were needed to investigated the protective therapeutic effects of Api m 1/microemulsion (66).

We highlight the potential of nanosystems in skin permeation and allergens/adjuvants delivery. However, there is still much to be understood. For instance, the physicochemical properties of nanocarriers including size, shape, rigidity, and surface charge can interact with the biological components and affect skin penetration. In this sense, better understanding on their physicochemical properties is necessary for precise epidermal delivery. Moreover, the antigen presentation, maturation and migration, and induction of T-cell differentiation requires proximity between APCs and nanoparticles. The presence of nanoparticles can alter APC functions, which is essential for a well-oriented and effective immune response. Besides, only a few nanocarriers have been approved as pharmaceutical products, due to issues like large-scale synthesis, stability. Bringing a nanosystem for EPIT from bench to market remains a lengthy process.

## Conclusion

AIT remains the only disease-modifying treatment capable of achieving long-term desensitization in allergy patients. Although various AIT systems, such as SCIT and SLIT, have been clinically approved to treat respiratory allergies, their frequent local or systemic side effects, as well as the substantial time, monetary, and effort commitments of patients pose certain limitations. Skin-based AIT systems have emerged as an alternative option in many preclinical and clinical studies. While no EPIT platform has been FDA-approved up to date, numerous encouraging studies suggest that EPIT could be a future trend for both respiratory and food allergies. The Viaskin, precise laser epidermal system, microneedles and nano-drug delivery system can sufficiently deliver allergens into epidermis with minimal skin reaction and rewrites the immunological response. Furthermore, addition of adjuvants in EPIT represents another strategy to enhance efficacy and safety. Overall, the innovative delivery systems for EPIT will allow for convenient, non-invasive, and self-administrable modalities of treatment.

## Author contributions

All the authors participate in initial discussion and composition of the review. ZW wrote the first draft and summarized **Table 1**, **2**. LW summarized the characteristics of these innovative delivery systems in **Table 3** and **Figure 1**. WW provided useful suggestions and finalized the review. All authors contributed to drafting this review and approved its final version.
